# STITCH enabled molecular interaction of glycrhizzin with peri-implant microbiota

**DOI:** 10.6026/97320630019499

**Published:** 2023-04-30

**Authors:** Karthickraj Selladurai, Sahana Selvaganesh, Vishnu Priya Veeraraghavan, Thiyaneswaran Nesappan, Rajalakshmanan Eswaramoorthy

**Affiliations:** 1 Department of Implantology1, Saveetha Dental College and Hospitals, Saveetha Institute of Medical and Medical and Technical Sciences, Saveetha University, Chennai 600077, India; 2 Department of Biochemistry, Saveetha Dental College and Hospitals, Saveetha Institute of Medical and Medical and Technical Sciences, Saveetha University, Chennai 600077,India; 3 Department of Biomaterials, Centre of Molecular Medicine and Diagnostics (COMManD), Saveetha Dental College and Hospitals, Saveetha Institute of Medical and Medical and Technical Sciences, Saveetha University, Chennai 600077, India

**Keywords:** Glycyrrhizin, *in vitro*, prediction, peri-implant microbes, peri implantitis

## Abstract

Glycyrrhizin is present in the root extract of the licorice plant used for various conditions, including gastric ulcers. Therefore, it is of interest to document the STITCH enabled molecular interaction of glycrhizzin with peri-implant microbiota. The peri implant pathogens includes *Aggregatibacter actinomycetemcomitans* (D7S-1), *Centipeda periodontii*, *Campylobacter gracilisi*, *Fusobacterium nucleatumi*, *Slackia exigua, Prevotella intermedia*, *Tannerella forsythia*, *Staphylococcus aureus*, Bacteroides fragilis, and *Bacteroides fragilis*. Hence, a user-defined query was used to conduct analysis on the provided bacterial strains whose molecular data available in the STITCH database. Thus, we used the STITCH tool to examine protein interactions and the VirulentPred tool to assess pathogenicity using the known molecular data on glycrhizzin and peri implant pathogens. Data shows that glycyrrhizin interacts with peri implant pathogens.

## Background:

Dental implants are prostheses with close resemblance of a natural tooth. The survival rates of implants are also about 96.4% to 99% [1] over a period of 10 years. The primary concern is the healing period, which is quite longer than fixed prosthesis. Maintenance is also a major concern for the survival of the implant. A major concern for the implant's survival [2, 3, 4, 5, 6, 7, 8, 9, 10, 11, 12, 13] is the health of the surrounding soft tissues, and progress of peri-mucositis to peri-implantitis. Therefore, it is of interest to determine whether glycyrrhizin use will have any effect on the microbes that cause peri implantitis.

## Materials and methods:

## Study design:

It is of interest to study the interaction of glycyrrhizin with *Aggregatibacter actinomycetemcomitans* (D7S-1), *Centipeda periodontii*, *Campylobacter gracilisi*, *Fusobacterium nucleatumi*, *Slackia exigua, *Prevotella intermedia**, *Tannerella forsythia*, *Staphylococcus aureus*, *Bacteroides fragilis*, and *Bacteroides fragilis* using known molecular data. Hence, a user-defined query was used to conduct analysis on the provided bacterial strains whose molecular data available in the STITCH database [13].

## Prediction of bacterial protein and metal oxide interactions:

The STITCH database (Version 5) is an open-source platform with an extensive collection of data about interactions, both physical and functional associations made possible by computational prediction of interactions from primary databases, the repertoire of proteins which interact with *A. actinomycetemcomitans* (D7S-1), *B. fragilis* (ATCC 25285), *C. gracilis* (RM 3268), *F. nucleatum* (ATCC 43037).14

## Prediction of subcellular localization of the virulent protein:

Cell surface proteins are of particular interest because they can be used as novel drug targets. Subcellular localization of proteins aids in the identification of drug targets and could serve as a potential target for new medications. An algorithm called Gneg-mPLoc uses an amino acid sequence to determine the likely location of a protein [14, 15].

## Prediction of subcellular localization of the virulent protein:

Cell surface proteins are of particular interest because they can be used as cutting-edge drug targets, and subcellular localization of proteins aids in the identification of drug targets and could serve as a potential target for new medications. Using a given amino acid sequence, the algorithm Gneg-mPLoc predicts the likely location of a protein's localization. [16]

## Results:

**Figure 1:**
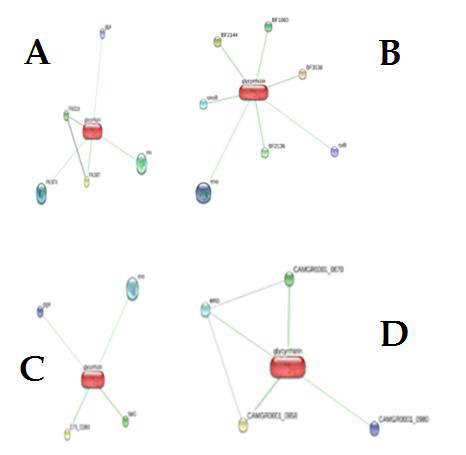
Interactions of glycyrrhizin with various proteins of (A) *Enterococcus faecalis*; (B) *Treponema forsythia*; (C) *Treponema. Denticola*; and (D) *Porphyromonas gingivalis*

The STITCH v5 tool was used to assess the interaction between the microbe and the element of interest. The protein target derivatives of the reactions were then further processed with the VICMpred and Virulentpred algorithms. It has been discovered that the molecule glycyrrhizin interacts with proteins crucial to cellular metabolism and other processes. It is intriguing to note that the element of interest also interacted with the peri-implant pathogens' virulence factors. The enzymes responsible for free radical production, glutaminase, glutamine synthetase, and the superoxide group of enzymes were involved in most interactions. The ten virulent factors' subcellular localization and epitope analysis were also performed in addition to these predictions.(Figure 1)

## Discussion:

Initial colonization of peri-implant surfaces by bacteria can happen in a matter of 2 weeks [17], the microflora on initial examination was thought to be similar to that of periodontitis more specifically the red complex bacteria and reports state that there is a difference in the total bacterial load between peri-implant biofilms and peri-implant mucositis. According to recent studies, the peri-implant biofilm is a complex ecosystem made up of a variety of rather variable. Based on the evidence currently available, the most prevalent species that have been identified are *Porphyromonas gingivalis*, *Tannerella forsythia*, *Treponema denticola*, *Aggregatibacter actinomycetemcomitans*, *Prevotella intermedia*, *Fusobacterium nucleatumi*, *Campylobacter* species, and Bacteroides species. Because of constant contact with tissues and body fluids, which act as a source for electrochemical interactions, mechanical loading of the implant leads to loss of ions by friction and electrochemical exchange, a process known as bio tribocorrosion It has also been suggested that long-term accumulation of biofilms and mechanical strain causes implant surfaces to degrade. Presence of high levels of dissolved titanium was detected in submucosal plaque around implants when compared to intervention free sites, thus indicating an association between Ti dissolution and peri-implantitis .The oxide corrosion products were found in newly formed trabecular bone and peri-implant vasculature and were systemically distributed. The oxide particles are cytotoxic, having an effect on immune cells, and it has been noted that the smaller the particle size, the greater the toxicity. Other researchers have observed peri implantitis in situations where the microbial threat is removed or controlled through frequent supportive measures. There is evidence that titanium oxide debris causes immune-modulatory changes that cause degenerative changes in osseous and periodontal tissues. This is due to the fact that immune cells around the implant, such as poly morpho nuclear neutrophils, macrophages, and monocytes, recognize titanium oxide debris. However it is interesting to note that TiO2 has a continuous photocatalytic antimicrobial activity against pathogens. Metal oxide alone or in combination with other metals like silver, copper or zinc is shown to have antimicrobial property and the same has been explored to a lesser extent In the present study we observe a good number of interactions between TiO2 and common peri-implant pathogens the target was mostly enzymes involved in cellular nitrogen metabolism which in turn brings about alteration in protein synthesis hindering the ability of bacteria cause virulence , thus it can be taken that titanium modifies peri implant microbiome and has potential antibacterial activity much light has to be shed on this aspect and the same would be clinically useful in management of peri implant disease by modifying implant surfaces or and we can deduce that peri implantitis is a complex disorder which has multifactorial causation, and more experimental exploration on this aspect to be carried out to produce effective treatment outcomes.

## Conclusion:

Data shows that the glycrrhyzin has virulence features against the protein of the oral microbes. This shows that glycyrrhizin have degenerative features. Thus, it has the potential to modify the peri implant microflora by interfering with their metabolic processes and could potentially increase the auto-immune response.

## Author contribution:

The first author (S Karthickraj) performed the analysis and interpretation and wrote the manuscript. The second author (Sahana Selvaganesh) contributed to conception, data design, analysis, interpretation and critically revised the manuscript. Third author (N Thiyaneswaran) critically reviewed the manuscript. All the authors have discussed results and revised the manuscript.
